# Building Community Resilience Through Trauma-Informed Solutions: Lessons Learned from a Social Accelerator in Rural North Carolina

**DOI:** 10.1007/s10597-024-01435-8

**Published:** 2025-02-13

**Authors:** Alison M. Elliott, Bethany Jana, Thi Vu, Macie Rush, Aaliyah Belk, Dane Emmerling, Vichi Jagannathan, Seth Saeugling, Abigail Hatcher

**Affiliations:** 1 Department of Health Behavior, Gillings School of Global Public Health, University of North Carolina at Chapel Hill, Chapel Hill, NC USA; 2 Center for Cancer Prevention & Control Research, University of California Fielding School of Public Health, Los Angeles, CA USA; 3 Department of Social and Behavioral Sciences, School of Public Health, Yale University, New Haven, CT USA; 4 Department of Health Equity, Gillings School of Global Public Health, University of North Carolina at Chapel Hill, Chapel Hill, NC USA; 5 Rural Opportunity Institute, Area L AHEC, Rocky Mount, NC USA; 6 Faculty of Health Behavior, University of the Witwatersrand, Johannesburg, South Africa

**Keywords:** Adverse childhood experiences, Community mental health, Rural health, Resilience, Social accelerator, Trauma

## Abstract

Adverse childhood experiences and intergenerational trauma are associated with a variety of negative health outcomes and are particularly prevalent among people of color and those living in rural communities. The social accelerator model offers a novel means of assisting organizations in scaling up their initiatives and increasing their impact. This study investigates the lessons learned from adapting the social accelerator model to address community-level trauma and build resilience in a rural setting. We conducted qualitative interviews with program staff and the initial cohort of participants of the Resilient Leaders Initiative: a trauma-informed social accelerator designed for public institutions in Edgecombe County, North Carolina. Participating community organizations included a local high school, church, and courthouse. In-depth interviews (*n* = 23) explored strengths, challenges, and perceived outcomes of the program and were thematically analyzed. The program’s deep community ties generated strong relationships among the cohort and created a safe space for participants to address trauma. Those interviewed identified the virtual program’s flexible structure as a key strength, but also reported experiencing confusion around roles, outcomes, and program language. A shared passion for building community resilience among participants and staff served as a key motivator throughout the program. Findings indicate that a flexible environment, strong interpersonal relationships, and deep community roots are essential to facilitating the creation of trauma-informed solutions among community organizations via the social accelerator model.

## Building Community Resilience Through Trauma-Informed Solutions: Lessons Learned from a Social Accelerator in Rural North Carolina

Intergenerational trauma is a widespread and costly public health problem (Gilgoff et al., 2020; Overstreet & Mathews, 2011). Experiencing a traumatic event during childhood, known as an adverse childhood experience (ACE), is associated with a variety of negative health outcomes, including psychological disorders, risk behaviors, and developmental disruptions (Kalmakis & Chandler, 2015). Examples of ACEs include experiencing abuse or neglect, witnessing abuse of a family member, and facing bullying and discrimination (Alhowaymel et al., 2021). An analysis of the 2015–2017 Behavioral Risk Factor Surveillance System found that 60.9% of U.S. adults had experienced at least one type of ACE in their lifetime, and approximately one in six adults, or 15.6%, had experienced four or more types of ACEs (Jones et al., 2020).

Traditionally underserved populations in the U.S. face a higher prevalence of ACES, including people of color and those living in rural communities (Giano et al., 2020; Crouch et al., 2020). Compared to those living in urban areas, children in rural areas experience higher rates of many types of ACEs, including parental death, household violence, and economic hardship (Crouch et al., 2020). Research has identified the need for greater trauma-informed programs in the U.S. to prevent ACEs from occurring and reduce the negative consequences of existing trauma, particularly for those living in rural communities.

The field of trauma-informed care focuses on recognizing the symptoms and consequences of ACES and other forms of trauma, and then adopting practices to reduce re-traumatization in the population (Maynard et al., 2019). Without protections in place, public institutions in the U.S. intended to support at-risk individuals can be trauma-inducing themselves (Substance Abuse and Mental Health Services Administration, 2014). For example, public school systems often create system-wide privileges for white students, resulting in poorer academic outcomes for students of color (Merolla & Jackson, 2019). The U.S. criminal justice system also perpetuates systems of trauma, especially among communities of color. Black Americans face disproportionately higher rates of violent and harmful interactions with law enforcement, contributing to a range of increased negative mental health outcomes in this population (McLeod et al., 2020).

Despite the perpetuation of systems of trauma in the U.S., as well as the adverse health consequences these systems inflict on certain populations, there is a notable lack of initiatives that explore community-level approaches to mental health promotion and engage organizations outside of the healthcare sector (Castillo et al., 2019).

### The Social Accelerator Model

The social accelerator has recently emerged as a model for supporting businesses in scaling up their programs (Innovation Edge, 2022), and offers a potential means for implementing trauma-informed strategies at the organization level. The social accelerator model first emerged in the tech start-up industry and involves improving access to resources for social entrepreneurs through relationship building, knowledge sharing, and investment (Causeartist, 2023). The model aims to assist participating organizations in growing their businesses and increasing the impact of their initiatives. Outputs from social accelerator programs range from vaccine distribution databases, to climate-based policy campaigns, to a digital assistant for healthcare workers (Fast Forward, 2021, 2022). While social accelerators typically serve start-ups and technology-based organizations (Barrehag et al., 2012), recent accelerator initiatives have explored how these programs can assist public and community-based organizations in establishing practices and policies to promote health and well-being.

### The Resilient Leaders Initiative and Edgecombe County, North Carolina

In 2021, Rural Opportunity Institute (ROI) developed the Resilient Leaders Initiative (RLI), a social innovation accelerator designed to assist rural public institutions in implementing trauma-informed practices in Edgecombe County, North Carolina. Edgecombe County is a majority-Black (57.3%) (U.S Census Bureau, 2019), rural community that faces a number of trauma-related health inequities, with 37% of children living in poverty, compared to 21% in the state of North Carolina (Edgecombe County Health Department, 2021). In 2019–2020, the incarceration rate in Edgecombe County was 410.4 per 100,000 people, compared to 304.2 per 100,000 people across North Carolina (Edgecombe County Health Department, 2021).

Traditionally, the largest employers and institutions in southern rural communities such as Edgecombe County are public agencies, e.g. public schools, law enforcement, health services, and government. RLI was created with the goal of supporting these community agencies in their efforts to address ACEs and intergenerational trauma. To our knowledge, RLI is the first social accelerator program of its kind: adapted for rural public institutions and focused on healing trauma and building resilience, through the use of human-centered design and systems thinking approaches.

RLI was designed over an 18-month period in collaboration with local leaders and drew from existing social accelerator best practices. During program development, ROI assembled local stakeholders to inform the program from the perspective of Edgecombe County residents. This group consisted of students and staff from public schools and a community college, church congregation members, non-profit organization leaders, healthcare workers, social service providers, and staff of the county police department. The group defined key areas of need in the community and assisted in program design.

RLI’s first cohort consisted of four teams, each from a different government institution or community organization in Edgecombe County, including a local high school, church, and courthouse. The accelerator spanned from March to November 2021 and was conducted virtually via Zoom due to the COVID-19 pandemic. Participants spent the first four months of the program understanding their place in an existing community-built systems map. This map identified patterns and forces that were leading to unaddressed trauma in the county, through a collaborative process that engaged over 400 community members (Vu et al., 2022).

**Fig. 1:**
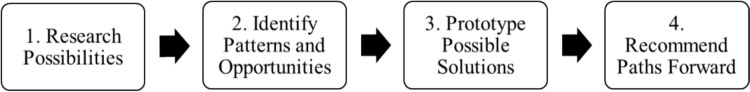
RLI Program Structure

Participants took part in a design challenge to develop and test trauma-informed practices within their respective organizations. Teams tested their ideas through an iterative process called “little bets” as they searched for effective ways to prevent and address trauma in the community. First, participants identified a target population currently underserved by their agency, based on data and qualitative interviews. Participants then used human-centered design methods to understand opportunities for innovation within their organization, based on their users’ experiences. This process culminated in participants co-designing “little bet” tests to see where they could create a measurable impact and reach their target population (see Fig. 1).

For example, one participating organization accomplished their “little bet” and became the first public alternative learning program in the state of North Carolina to successfully bill mental health services to Medicaid. This process built a sustainable funding source to hire a licensed clinical social worker to provide services to their highest-need students, at no additional cost to the school district. Other “little bets” tested and implemented by the RLI cohort included evidence-based mindfulness circles to promote healing and stress management, connections to local businesses for internships and job skills programs, and a photovoice project to build social-emotional skills.

This article presents a detailed overview of the challenges, strengths, missed opportunities, and perceived outcomes of RLI, as reported by the first cohort of the accelerator program. Best practices for adapting a similar social accelerator program in other rural communities are also highlighted.

## Method

### Participants and Setting

This qualitative study occurred from August 2021 to April 2022 and was led by a team of graduate student consultants at the University of North Carolina at Chapel Hill Gillings School of Global Public Health, who partnered with ROI from August, 2021 to May, 2022. Twenty-three qualitative interviews were conducted with RLI participants (*n* = 7, 41% of 17 total), coaches (*n* = 5, 100% of 5 total), facilitators (*n* = 7, 70% of 10 total), and RLI staff members (*n* = 4, 100% of 4 total).

RLI participants included representatives of rural public institutions in Edgecombe County. Contracted coaches served as participant teams’ primary point of contact during RLI, meeting with teams weekly, keeping track of team progress and supporting participants throughout the program. Also contracted, facilitators consisted of experts from around the country who joined the virtual monthly convenings and presented information related to their area of expertise (i.e. data and evaluation, anti-racist practices, human-centered design). RLI staff developed the curriculum for RLI, hired coaches and facilitators, led recruitment of participants, and supported the implementation of the program.

### Data Collection

RLI participants, coaches, facilitators, and staff were interviewed virtually via Zoom. Semi-structured interview guides were developed and focused on exploring program characteristics and processes, as well as strengths and challenges. Interviewees were asked to suggest strategies on how RLI could be improved for future cohorts and share their long-term hopes for RLI.

### Data Analysis

Each interview was digitally recorded and transcribed. Transcripts were de-identified and labeled by participant type (i.e. participant, coach, facilitator, RLI staff). A thematic codebook outlining broad categories was developed using a deductive technique (Miles & Huberman, 1994). To ensure consistency in coding, several authors independently coded one transcript and then met to resolve any inconsistencies and make necessary adjustments to the codebook. After this discussion the codebook was finalized, and each transcript was individually coded by a member of the student team. The team then developed an analytical report for each of the broad themes/categories by reviewing the excerpts coded for that theme. This inductive technique involved a combination of individual and collaborative identification of more fine-level themes (Bernard & Ryan, 2010).

### Ethical Considerations

All interviewees received a copy of an informed consent document. Interviewers had no prior relationship with any of the interviewees. Interviewers reviewed this information with the interviewees and obtained verbal informed consent prior to each interview. Study procedures were approved by the Institutional Review Board at the ​​University of North Carolina at Chapel Hill (#22–2038).

## Results

Participants identified several themes when describing their experiences with RLI. These themes included: (1) tensions between flexibility and ambiguity, (2) benefits of being embedded within the community, (3) differences in perceptions of diversity, equity, and inclusion, (4) difficulties understanding language and jargon, (5) feelings of connection and support across the cohort, (6) challenges and strengths of the virtual format, and (7) contrasts between participant passion and burn-out.

### Tensions Between Flexibility and Ambiguity in the Program

A majority of interviewees highlighted the program’s flexible nature as a strength. They described RLI leadership as accommodating and taking an iterative approach when designing the program’s curriculum. RLI staff were seen as receptive to feedback from the cohort. Participants, facilitators, and coaches noted that RLI staff continuously adapted the material to better meet the needs of participating organizations. Many interviewees stated that RLI staff “walked the walk” when it came to creating a culture that encouraged and listened to feedback. With regard to the onboarding process, coaches and facilitators commented on the benefits of this iterative mindset:

I thought [the onboarding process] prepared us pretty well. I think that just having the expectation that this cohort’s going to be adaptive…we’re gonna have an idea of where we’re going, but there’s gonna be a lot that we’re figuring out between sessions based on how things are actually progressing with the teams. I think that having that expectation in advance really set us up well because we knew that we had to be flexible. (Facilitator)

In a few cases, teams described instances where their interests and goals shifted during the program. For example, one organization decided to switch their focus from mental health to homelessness midway through the program. Both a participant and coach from the team remarked that RLI staff fully supported this switch:

I was impressed with how RLI handled that. It wasn’t like a, “You’re outta the program. Don’t come to us for funding for that because you didn’t finish this project.” It was like, “If they want your support to do that for the rest of this time, then we’ll support that.” They were very flexible and adaptive, which I think is really important in building these relationships. (Coach)

Another coach reflected that RLI staff also allowed coaches to customize materials, stating:

I could coach the way I felt like I needed to coach and be responsive to the team…I feel like RLI, by giving me enough room to do my own thing, I didn’t feel overwhelmed like I was not doing what other people did. I was able to do it my way and honor the vision but show up in a way that was authentic for me. (Coach)

While interviewees described the benefits of RLI’s flexible structure, the open-ended nature of the program at times led to a lack of clarity on the roles of staff. Unclear expectations for coaches and facilitators led to teams feeling “a little restricted” and a desire to “utilize skills a little more effectively.” Some facilitators stated that while they were encouraged to take the lead with content planning and program delivery, this resulted in the feeling that there were “too many cooks in the kitchen.” Facilitators found it challenging to design a streamlined and cohesive program, especially given the time-constraints and lack of prior connection with other facilitators.

Confusion around expectations also corresponded with feelings of uncertainty around the intended program outcomes. Participants, coaches, and facilitators expressed uncertainty about what they were “supposed to be getting out of [RLI].” Some interviewees felt that as the program came to an end, there was a pressure to let the “systems strengthening piece fall away” and “just get a great project.”

To reduce this sense of ambiguity, a few interviewees suggested that RLI staff provide an overview at the beginning of the program. This overview could include a description of the content to be covered, an outline of the program process, expectations for different team members, and examples of success. One facilitator suggested incorporating a logic model, “being quite clear about what the outcomes are that you want at the other end. Then actually being quite prescriptive about how you’re gonna get there, and then plugging in our skills where necessary.” Interviewees also suggested having program alumni present their work and outcomes from RLI to future cohorts, providing current teams with a reference point throughout the program.

### Benefits of Being Embedded in the Community

ROI’s embeddedness in the Edgecombe County community increased the credibility and strength of the accelerator program. By taking the time to form relationships in the community, ROI established itself as a community-centered organization and laid the necessary groundwork before launching RLI. ROI was regarded positively for allowing the mission and content of its programs to be based on the self-determined needs of community members. Several interviewees viewed RLI’s community focus as something that distinguished the initiative from other programs they had worked with in the past. One facilitator stated, “it’s a very community-led initiative and organization. It’s not people parachuting in with tools and techniques, but building tools and techniques in community with those that are every day working to build a stronger social fabric in these areas.”

RLI’s community-centered mission also increased trust between participants and program leadership, as community members felt their outcomes were prioritized over any metrics:

The reason I trust Rural Opportunity is because I see the data, the results of the work, but I also see that they are not just about the numbers. They’re about the people…Lots of organizations just wanna know, “well, how many people? What’s the stats?” Rural Opportunity takes the time to get to know the why behind things and to get the nuance of what’s going on. (Participant)

This human-centered approach allowed ROI to further establish credibility among the community members involved in the accelerator and increase receptivity to the program.

### Differences in Perceptions of Diversity, Equity and Inclusion

When discussing diversity, equity, and inclusion (DEI), participants, coaches, and facilitators often described RLI’s atmosphere as a “safe space.” Coaches, facilitators, and participants viewed the program’s environment as welcoming and open, and felt comfortable sharing their experiences and learning from others without judgment. Participants noted that this positive environment stood out to them and was especially impressive compared to other initiatives they had participated in:

After going through RLI, where I always feel that I’m in a safe space no matter what we were doing, that was just astounding. There were other people there who also, I think, had been through RLI types of things, and we were just dumbfounded…RLI always made us feel safe, and it was very enlightening to be in that space and hear people share their points of view. (Participant)

When asked about what they felt RLI’s “secret sauce” was, another participant identified this safe space where equitable learning occurred regardless of education level:

It’s a safe space that you can have the PhD in all the things, or you can be completely ignorant in all the things. You show up, and everyone’s on the same, equal space. Everyone is gonna learn from where they are, and there’s equity in it. (Participant).

A few interviewees noted that RLI was strengthened by the diversity of the cohort, amongst whom participants could safely express their full identity, including race, religion, and sexual orientation. Participants felt that there were both people like them on the team that could relate to their experiences, as well as others from unique backgrounds that they could learn from.

We had a Black coach, and that was just helpful…It’s good to have people who understand those dynamics. I would also say that the plus overall, which I saw not just in our coach but in also the other presenters, some of them were Black. Some of them weren’t, but they all understood the dynamics of race in America…we didn’t have to overdo work or hide any perspective of ourself or anything like that in order to placate to her, in order to placate to others. (Participant)

In addition to expressing their own identities, participants also felt that there was room to learn about the diverse experiences of others without judgment. One coach explained that RLI staff, coaches and facilitators generated these conversations for individuals to learn and grow, in this case regarding the use of pronouns:

You can say, “I don’t really get pronouns.” Oh, you’re canceled. No. I wasn’t saying I’m against it. I’m saying I don’t understand it and I need to learn. We can jump to trying to be so super woke that we shut the people up who are trying to learn. RLI created a space where people could—it wasn’t a big part of it, but I think people could talk about these—the way that my team talked, they call it taboo topics. (Coach)

Although interviewees described positive experiences in what they felt was a “safe space,” at least one coach and one facilitator noted that RLI leadership could have better represented the community they were serving. The racial backgrounds of RLI staff, coaches, and facilitators were less representative than that of Edgecombe County, which is 57.3% Black (US Census, 2019). Among RLI leadership, 38% of facilitators were Black, 40% of coaches were Black, and 50% of RLI’s full time staff were Black. Although the coach and facilitator who raised this concern did not notice its impact on their relationships with participants, they admitted to wondering how participants felt.

I was surprised there were not more facilitators who represented the population, right. Other than the consultants that did the diversity and equity, from what I remember, and myself, all of the other facilitators were white, and not that we can’t learn things from everybody. However, I go back to my background in the school system and education. I need my Black students to see Black people in leadership. I need them to hear that voice, and that was not there. It was a little bit of a disconnect for me. (Facilitator)

Another facilitator also expressed concern about the fact that facilitators and coaches were not based in rural areas or in the community:

Many of the coaches and facilitators were not based in [county] and the participants were. It seemed like we had a good working relationship — I just wonder if anybody—how any of the participants felt about that tension…between outsiders and insiders, and outsiders in terms of geography, outsiders in terms of race. (Facilitator)

RLI staff shared that they made intentional choices to hire both people with local connections and those located around the country. They made this choice based on RLI’s design process, during which community members shared their desire to learn from experts and those with experiences beyond the local community. For future cohorts, coaches and facilitators made two suggestions to address DEI. The first was to recruit a set of coaches and facilitators that better represent the racial makeup of the community. The second was to have RLI alumni serve as coaches or peer supporters.

### Difficulties Understanding Language and Jargon in Program Content

Multiple participants identified difficulties understanding the language used in the program’s curriculum. Several participants unfamiliar with program concepts—such as systems mapping or systems thinking—felt that they were behind and needed to catch up. The concepts seemed too “academic,” “too complex”.

It can seem overwhelming…In some of the presentations…if you’re not familiar with the vernacular, you’re not familiar with the academic language, then you can get lost…people were having these conversations, and they’re using these academic terms. A lotta people have no idea what that stuff means. They have no idea what you’re talking about. (Participant)

Coaches and facilitators also expressed difficulty getting on the same page when sharing academic information with participants. Facilitators and coaches worried about overwhelming participants and wrestled with how to best present information. One facilitator described “tensions around how to approach a process” among the facilitators.

Despite initial difficulties understanding jargon, participants appreciated the efforts of program leadership to break down confusing material by reiterating key points and adjusting language. Multiple participants highlighted instances of presenters rewording program content after receiving feedback that it was difficult to follow.

I am not a mental-health-trained person. Some of the conversations at the very beginning were overwhelming to me because I didn’t know what they were talking about. I didn’t get the language. I’m like, “I don’t know what y’all talking about. Y’all over my head. You’ll have to bring this down to layman’s terms so we can understand.” They were very receptive of that, and I really appreciated that. (Participant)

This process of “breaking down” program content allowed initially struggling participants to be more at ease with the material and process key concepts better.

### Feelings of Connection Across the Cohort

A majority of interviewees identified opportunities to collaborate with other members of the RLI cohort as a strong point of the program. They described a sense of community across the participating organizations. This sense of connection with others in the cohort provided a new network of support for teams’ work. As explained by one coach:

I think having the cohort itself is a strength. You have strength in numbers. You’ve got folks coming together on a monthly basis. While you’re doing your own thing in terms of programming, you’ve got this larger mission that you’re all trying to forward. (Coach)

Participants also described a new awareness of others in their field. They felt as though they were not alone in their work to address community trauma and appreciated learning from others in the cohort.

I feel that now I’m better equipped to handle challenges because I know that I have a friend in the community, or resource in the community, that can help me navigate some things or some challenges and connect me with people. (Participant)

Teams benefitted from hearing about the ways others dealt with issues and problem-solved during the program. One participant felt that in break-out rooms with members of other teams, “you got a chance to hear and experience what the other teams were working with and some of the issues, some of the traumas that we were going through you would hear of in different groups. It helped.**”** By creating a shared community across the RLI cohort, members could openly exchange ideas and assist one another as they created trauma-informed solutions. A coach described this process of teams learning from one another:

As we all kind of got to know each other, and all of these monthly relationships grew, I think people were pretty open about saying, “Well, I don’t understand this, or make sure I’m doing this the right way, or what about this?” (Coach).

While a sense of community among the cohort developed throughout the course of the program, the majority of those interviewed desired a greater emphasis on relationship-building over the course of RLI. When asked about suggestions for future cohorts, participants often wished for more opportunities to connect with others across the program. One facilitator expressed, “it’s such a great group of really experienced and talented people. I think there were ways that we could’ve been maybe a bit more interconnected across the project.” Similarly, a coach reflected that teams would have benefited from “heavy relationship building, I think would’ve been a wise investment earlier on.”

Coaches and facilitators also expressed wanting greater connection with each other. One coach said:

I feel like if I had stronger relationships with the facilitators and other coaches, that maybe I would have known or even felt more comfortable to call on them for additional support. Everyone offered it. Everyone was always like, “I’m here too”- but I didn’t really know what to call on them for. (Coach)

While interviewees were enthusiastic about the new network of support they had gained from the program, allocating greater amounts of time to foster these relationships may have reduced any feelings of missed opportunities.

### Challenges and Strengths of the Virtual Format of the Program

As a result of the COVID-19 pandemic, interviewees described general feelings of the ease and convenience that came with RLI’s virtual setting. However, the remote setting also resulted in a desire for deeper connections, as interviewees felt it was more difficult to build relationships without in-person meetings. One facilitator reflected:

Because I’m a social person, I felt stifled…COVID has definitely been a hindrance to that… I would have loved for us to been able to do it in person, because that connection piece gets missed and gets lost when you’re on Zoom. (Facilitator)

Coaches also expressed feelings that they could have better led their teams had the program been in-person, and some participants preferred to do activities in-person.

I like classroom stuff with posters and sticky notes, but it was still good. I think I’m not the only one who sometimes got Zoom burnout, but it was good to still be able to meet. I think being in a room setting was always better. (Participant)

A few participants were less comfortable working with technology. One RLI staff member noted having internet issues, “everyone can’t have high-speed internet. The internet that you do have is shaky.” The virtual format added to existing feelings of missed opportunities for connection among the cohort.

### Contrasts Between Participant Passion and Burnout

Facilitators, coaches, and RLI staff members were all impressed with the enthusiasm and commitment of the program’s participants to addressing trauma in their community. They described being “blown away” by participants’ passion, which stood out from other groups they had worked with. One facilitator described how impressed they were with the drive of the participants:

They were so gracious and so kind, and clearly cared so much. Yeah, it’s definitely a different type of group than I’m used to working with…people would be, like, “Oh my God, it’s God’s calling to do this,” it’s, like, wow, I’ve never heard that at work, you know? It’s definitely a very different side than I’ve gotten. Much more, I don’t know, just maybe sincere and kind. (Facilitator)

Participants also described the excitement they felt towards the program and their cohort:

A lot of it worked naturally because we would just sit back and talk to one another and just the excitement of getting things done, what kind of ideas we could come up with, and when the next meeting would come up, we would just have ideas of what we wanted to do, and would bring them to our coach, and she was spot on, and she was ready to just go forth with it. (Participant)

However, when asked about challenges, interviewees expressed that they “worked harder in the beginning” and felt “less energy” and “less motivation” as the program went on. They pointed to “the nature of the stressors we’re all living under” as a contributor to burnout. To avoid this, interviewees suggested shortening the length of convenings, communicating expectations upfront, and assigning less homework.

## Discussion

This study identified strengths, challenges, and lessons learned from a social accelerator program to address trauma and build resilience in a rural context. To our knowledge, RLI is the first social accelerator program in the U.S. both rooted in a rural community and focused on engaging public institutions. Data from our qualitative interviews suggest that this social accelerator model can aid rural public institutions in implementing trauma-informed practices.

The social accelerator model presents a unique approach to addressing community-level trauma, through the creation of a flexible environment tailored to the needs of participants. RLI staff’s receptiveness to feedback and willingness to revise program content was repeatedly highlighted across interviews as a key benefit of the program. By harnessing the existing assets of participating organizations and allowing participants to determine their own goals and desired outcomes, the accelerator prioritized the experience of participants rather than prescribing certain solutions.

This community-first nature of the accelerator model aligns with recommendations from both the fields of trauma-informed care and rural health: that initiatives should work with existing leaders and leverage resources to meet community needs and foster resilience (Afifi et al., 2022; Ellis & Dietz, 2017). The American Academy of Pediatrics highlights cross-sector collaboration between health care systems and public agencies in their policy recommendations for integrating trauma-informed care (Duffee et al., 2021). The sense of connection felt by participants, both with staff and other members of the cohort, suggests that social accelerators can build networks and support systems not only among tech start-ups, but also community organizations seeking to address ACEs and other forms of trauma.

While social accelerators offer a promising approach for addressing community-level trauma, our findings also demonstrate areas for improvement and increased clarity in this model. Confusion around roles and intended outcomes may be an unintended consequence of the participant-centered nature of the social accelerator model, consistent with community models in other fields (Ortega et al., 2018; Stewart et al., 2019). As social accelerators develop further, particularly in trauma-informed research, programs should incorporate tools such as logic models, to not only encourage participant engagement but also bolster the program’s structure. These models can be iterative and revised as participant needs change (Afifi et al., 2011; Peyton & Scicchitano, 2017; Rehfuess et al., 2018).

A trusting relationship between community members and RLI staff proved to be a key factor to program success. ROI’s pre-existing and deep roots in the community created a strong foundation that accelerated buy-in from participating institutions. The incorporation of human-centered design and systems thinking models into the program, as well as the iterative nature of the program’s curriculum, furthered the trust between participants and RLI staff. In understanding that RLI staff shared the community’s interests and goals, participants felt comfortable discussing topics that may otherwise have been taboo and voicing their needs when issues arose.

The crucial role of these interpersonal connections in RLI’s discussions around community-wide trauma adds to the existing literature on the relationship between feeling that one is in a “safe space” and improved psychological outcomes (Andriessen et al., 2022; Bluth et al., 2023; Campbell et al., 2004). Edmonson describes the benefits of “psychological safety” in the workplace, when individuals feel that they may speak vulnerably without facing negative consequences. This feeling of safety subsequently allows employees to be their true selves, leading to better workplace outcomes and insights (Edmondson, 2019). Trauma-informed care research has found that interventions that increase a community’s feelings of connection can reverse the sense of isolation and mistrust that often results from collective trauma, after events such as natural disasters (Somasundaram, 2014). The benefits of trust are also observed at the provider level, with trusting relationships between patients and mental health professionals serving as a key factor for improving patient outcomes and preventing the re-traumatization of patients (Sweeney et al., 2018). Our findings similarly suggest that fostering deep relationships throughout a community-based program allows participants to feel psychologically safe and address problems as complex as intergenerational trauma.

While the trusted relationships between RLI staff and community organizations enabled success, interviewees still identified areas of the program that could have better met community needs. In connecting community members with outside resources, certain aspects of RLI felt less tailored to participant projects and challenged the essential trust within the program. Facilitators remarked on how, at times, they felt like outsiders due to not being from the community. Interviewees described a desire for greater representation of community members, specifically people of color, among program leadership: a common critique of community building programs (Ali et al., 2023; Quinn, 2004; Stewart et al., 2019). Bringing in these outside experts may have contradicted the program’s mission of being rooted within the community.

Although coaches’ and facilitators’ willingness to respond to this feedback and adjust content may have maintained the trusted relationships essential to the program’s success, greater incorporation of community voices at every step of the process could have increased the cultural competency of the program and prevented these challenges from arising at all. The suggestion from coaches and facilitators that RLI alumni serve as mentors for future teams could further integrate the program in the community and reduce confusion. Additionally, allowing alumni to have a voice in curriculum design might decrease language barriers. Future programs addressing community trauma should not only adopt a flexible and iterative nature like RLI, but also recruit leadership with backgrounds more similar to that of the community.

Findings from this evaluation of RLI also highlight the impact that virtual settings hold on relationships across the social accelerator model. As the first cohort matriculated through the COVID-19 pandemic, some of the difficulties of participating in this program virtually included slower internet connection and missed opportunities to meet people in-person. On the other hand, participants and staff appreciated the greater flexibility offered by the program’s virtual format, a shift that has been supportive in other community-based health work (Irish et al., 2021; Shah et al., 2021). Participants reported feeling a strong connection to their cohort despite the limitations of virtual programming, supporting evidence that deep relationships can bolster program results even in virtual settings (Bluth et al., 2023; Wilson et al., 2022).

Virtual programs possess the unique ability to reach underserved populations, including rural communities, whether due to geographical barriers or a global pandemic (Khairat et al., 2019; Marcin et al., 2004). The findings from our study suggest that efforts to expand virtual programming, especially in the field of trauma-informed care, should prioritize creating a sense of community and relationship-building, to overcome feelings of disconnect that can arise from not conducting programs in-person. These results are consistent with findings that virtual initiatives can improve outcomes when they increase participants’ sense of support. For example, the creation of the National Nursing Home COVID-19 Action Network increased emotional and social support among nursing home staff during the pandemic and bolstered resilience in this population (Baughman et al., 2021).

These findings should be viewed in light of design limitations. First, positionality of the interviewers (the UNC student team) may be present in the findings. Although the student team conducting interviews were external to RLI, it is possible that the team’s relationship with ROI staff could have influenced interviewees to report more positive findings. In an attempt to prevent this bias, the interviewers reiterated their position outside of the organization to those interviewed. Nevertheless, findings should be interpreted in light of potential social desirability bias.

In addition to the positionality of the interview team, the potential selection bias of participants in RLI may pose another limitation to the study. RLI was designed to serve rural organizations who were already interested in “piloting new practices that lead to healing—and reimagining policies that have caused trauma and harm,” (Rural Opportunity Institute, 2021a, 2021b). As participating organizations were already invested in adopting trauma-informed practices, they plausibly had greater buy-in from the start of the program. This is not a methodologic shortcoming, as qualitative research rarely aims for generalizability, but it does offer lessons learned from among a highly engaged group as opposed to general community representatives.

Despite these limitations, this study illuminates several best practices for adapting a social accelerator model to implement trauma-informed practices in rural community agencies, including creating a flexible but clear program structure for participants, fostering strong interpersonal relationships and deep roots in the community, and creating a safe space for participants of all identities. The social accelerator model offers an iterative and adaptive framework for addressing community-level trauma. In this case, the community-centered attitudes of the accelerator’s staff allowed the program to have credibility amongst organizations and served as the foundation to solving any conflicts that arose during the program. Future research should explore how adapting a social accelerator model can be leveraged to address ACEs and heal trauma within different rural community contexts and the downstream effects.
